# Liver Biopsy for Histological Assessment – The Case Against

**DOI:** 10.4103/1319-3767.61244

**Published:** 2010-04

**Authors:** Faisal M. Sanai, Emmet B. Keeffe

**Affiliations:** Hepatology Unit, Department of Medicine, Riyadh Military Hospital; Liver Disease Research Center – King Saud University, Riyadh, Saudi Arabia; 1 Division of Gastroenterology and Hepatology, Department of Medicine, Stanford University Medical Center, Stanford, CA, USA

**Keywords:** Biomarkers, FibroScan, FibroTest, histology, liver biopsy, noninvasive assessment

## Abstract

Percutaneous liver biopsy (LB) remains an important tool in the diagnosis and management of parenchymal liver diseases. In current practice, it is most frequently performed to assess the inflammatory grade and fibrotic stage of commonly encountered liver diseases, with the diagnostic role relegated to secondary importance. The role of LB remains a vastly controversial and debated subject, with an ever-increasing burden of evidence that questions its routine application in all patients with liver dysfunction. It remains, essentially, an invasive procedure with certain unavoidable risks and complications. It also suffers shortcomings in diagnostic accuracy since a large liver sample is required for an ideal assessment, which in clinical practice is not feasible. LB is also open to observer interpretation and prone to sampling errors. In recent years, a number of noninvasive biomarkers have evolved, each with an impressive range of diagnostic certainty approaching that achieved with LB. These noninvasive tests represent a lower-cost option, are easily reproducible, and serve as suitable alternatives to assess hepatic inflammation and fibrosis. This article aims to debate the shortcomings of LB while simultaneously demonstrating the diagnostic accuracy, reliability and usefulness of noninvasive markers of liver disease thereby making the case for their utilization as suitable alternatives to LB in many, although not all, circumstances.

Liver biopsy (LB) is an important diagnostic tool that assists determination of specific diagnoses and directs therapeutic decisions in patients with acute and chronic liver diseases. Over one hundred years ago, Paul Ehrlich introduced the procedure as a means of studying liver histology.[1] Since then LB has gained widespread acceptance for the assessment of liver abnormalities associated with many types of liver diseases. The popularity of LB was facilitated by the Menghini “one-second liver biopsy” technique,[2] which provides samples suitable for various morphological studies, including histochemical, immunohistochemical, ultrastructural and, more recently, molecular biology studies.

The examination of an LB specimen under the microscope is a direct way to identify changes in hepatic tissue and either make a specific diagnosis or determine the grade and stage of chronic liver disease. When it was initially developed, LB primarily served as a diagnostic aid to determine the etiology of liver dysfunction. However, with expansion of knowledge in relation to pathogenesis and natural history of various liver diseases, and the availability of more sensitive and accurate serologic, virologic, genetic and immunologic laboratory tests as well as radiographic techniques, the role of LB in clinical practice has undergone a major change. LB remains a key test to provide a diagnosis, especially in the presence of significant hepatic dysfunction and lack of diagnosis in spite of a comprehensive laboratory evaluation for viral, genetic and autoimmune diseases. In current practice, however, LB is most often performed to assess the degree of necroinflammatory and fibrotic changes, thereby providing essential prognostic information on which to base therapeutic decisions. LB has remained the “gold standard” mainly because of the absence of better alternatives.

However, at long last, substantial progress has been made to break the monopoly that LB has maintained on the evaluation of hepatic tissue. Alternatives to this invasive test have been proposed and are deemed to be as good as biopsy and less damaging to the patient, and include predictive tests for assessment of steatosis, inflammation and fibrosis.[3] Additionally, it has become apparent that LB, far from being a “gold standard,” is at best an imperfect standard that has attracted criticism over its general application. Increasing evidence challenges the notion of LB as the reference against which all other techniques must be measured.[4–10] Its role remains a controversial subject, and an ever-increasing number of authors have questioned the need for its routine application in all patients with liver dysfunction.[11–13]

Simultaneously, evidence has accumulated promoting the use of noninvasive means of assessing liver histology. While investigators initially focused on a combination of laboratory tests such as reversal of aspartate aminotransferase (AST)/alanine aminotransferase (ALT) ratio or AST/platelet ratio index (APRI), more recently there has been a concerted effort to identify novel markers of fibrosis, necroinflammation and steatosis.[14–18] A recent systematic review of noninvasive biomarkers by Poynard *et al*[19] identified a total of 2237 references between 1991 and 2008 to novel biomarkers of liver fibrosis, of which 14 have been validated. This clearly represents an escalating interest in the study of noninvasive markers of liver disease.

The initial international guidelines, consensus statements and expert panel opinions on the management of chronic viral hepatitis were unanimous in their recommendation of LB for pretreatment evaluation of the disease.[20–26] However, more recently, the European Association for the Study of the Liver guideline statement for the management of chronic hepatitis B (CHB) published in 2009 supports the use of noninvasive markers for disease stratification, providing credibility to their reliability and reproducibility.[27] Moreover, the use of such tests is rapidly evolving in practice. A recent survey of 546 hepatologists in France revealed that 81% used the noninvasive biomarker FibroTest–ActiTest (Biopredictive, Paris, France) and 32% used transient elastography, resulting in an impressive reduction in the use of LB by 50% for chronic hepatitis C (CHC) patients.[28]

In this article we will discuss the shortcomings of LB. In the same vein, we aim to demonstrate the diagnostic accuracy, reliability and usefulness of noninvasive markers of liver disease and make the case for their utilization as suitable alternatives to LB in the evaluation of chronic liver diseases.

## LIVER BIOPSY

### Complications of biopsy

Percutaneous LB is an invasive procedure and is associated with a significant risk of complications. These complications may vary from minor events, such as pain and transient hypotension, to major complications, including (i) hemorrhage (intraperitoneal, intrahepatic, hemothorax); (ii) puncture of viscus (gallbladder, colon, pleura); (iii) inadvertent biopsy of the kidney or the pancreas; and (iv) intrahepatic arteriovenous fistula formation.[29,30] Significant bleeding and bile peritonitis are serious complications and may lead to mortality [Table 1]. The mortality rate from LB is reported to range from 0.01% to 0.1%.[30,31]

**Table 1:** Noninvasive markers of liver histological assessment including tests for fibrosis, necroinflammation and steatosis

**Test**	**Components**	**Liver disease**
FibroTest-ActiTest	Age, sex, A2M, GGT, haptoglobin, total bilirubin, apolipoprotein A1, ALT	HCV, HBV, NAFLD, ALD
FibroSpect II	HA, TIMP -1, A2M	HCV
ELFGA	Age, PIIINP, HA, TIMP-1	Mixed
FibroMeter	Age, sex, A2M, HA, platelet count, AST, prothrombin	Mixed
HepaScore	Age, sex, HA, A2M, GGT	HCV
APRI	AST, platelet count	HCV, HBV
FibroIndex	AST, platelets, gamma globulins	HCV
AST/ALT ratio	AST, ALT	HCV, HBV
Forns index	Age, platelet count, GGT, cholesterol	HCV
Pohl score	AST, ALT, platelets	HCV
AP index	Age, platelet count	HCV
CDS	AST, ALT, platelet count, INR	HCV
FPI	Age, AST, cholesterol, past alcohol use, insulin resistance	HCV
SteatoTest/NashTest	BMI, AST, serum glucose, triglycerides and cholesterol, age, sex, A2M, GGT, haptoglobin, total bilirubin, apolipoprotein A1, ALT	NAFLD
FibroScan	Hepatic transient elastography	HCV, HBV, PBC, PSC, ALD
SHASTA index	HA, AST, albumin	HCV
BAAT score	BMI, age, ALT, triglycerides	NAFLD
NAFLD fibrosis score	Age, BMI, platelet count, albumin, AST/ALT ratio, IFG/diabetes	NAFLD

ALT= Alanine aminotransferase; AST= Aspartate aminotransferase; GGT= Gammaglutamyl transpeptidase; HA= Hyaluronic acid; A2M= Alpha-2-macroglobulin; INR= International normalized ratio for prothrombin time; BMI= Body mass index; PIIINP= Amino-terminal propeptide of type III collagen; NAFLD= Nonalcoholic fatty liver disease; HCV= Hepatitis C virus; HBV= Hepatitis B virus; ALD= Alcoholic liver disease; PBC= Primary biliary cirrhosis; PSC= Primary sclerosing cholangitis; APRI= AST to platelet ratio index; FPI= Fibrosis prediction index; ELFGA= European liver fibrosis group algorithm; AP index= Age platelet index

It is significant to note that while pain is dismissed as a trivial complication, it is experienced in 84% of individuals during LB,[32] is severe in 20% and may persist beyond the day of procedure.[32] The pain immediately following LB can be highly distressing and a major ordeal for patients, thereby serving to discourage future LB. A recent French survey of 1177 general practitioners showed that 59% of patients infected with hepatitis C virus refused LB, an opinion concurred by 22% of the general practitioners.[33]

Likewise, LB-induced bleeding is often asserted to be an extremely rare complication although major bleeding is reported in up to 4.5% of procedures.[34] In fact, the most common cause of death associated with LB is due to bleeding, which may occur in up to 1.6% of patients undergoing the procedure.[34] Various predictors of bleeding have been identified (coagulopathy, multiple passes, cirrhosis, tumor), and while extreme precautions are taken to avoid biopsy in the face of abnormal coagulation, most bleeding cases (>90%) occur with an international normalized ratio (INR) less than 1.3.[35–39]

LB is frequently cited as a simple procedure that may be performed safely at the bedside by relatively junior personnel. As an ever-increasing number of LBs are being performed for a widening spectrum of indications, LB may more often be performed by less skilled individuals. In a study demonstrating the relevance of the learning curve, major morbidity (4.7%) and mortality (2.2%) both arose in the setting of personnel inexperience.[34] Similar studies in the past have shown that complication rates are markedly higher when the procedure is performed by less-experienced individuals.[39,40] Thus, it is anticipated that an inordinate number of complications of LB may arise in the foreseeable future. Given these complications, there is an understandable reluctance on the part of patients to undergo repeated biopsies that may be required to monitor disease progression, especially in the context of antifibrotic therapy development.

Finally, much has been made of the transvenous approach to LB as a means of reducing serious complications. In a recent systematic review of 7649 transjugular LB, minor and major complications were reported in 6.5% and 0.6% of interventions, respectively, along with an accompanying mortality rate of 0.09%.[41] Similarly, ultrasound guidance is unlikely to reduce the complication rate of LB, since imaging fails to identify small intrahepatic arteries, which are the usual causes of serious bleeding.[42] In deference to this rationale, data from a retrospective study showed that in biopsies performed with ultrasound guidance, the risk of major hemorrhage was somewhat higher than nationally published figures.[43] This suggests that, as yet, there are no definitive means of avoiding the usual major complications of LB.

### Inadequacy of biopsy specimens

A number of studies have shown that sampling errors occur when the samples obtained from a target population (or tissue) fail to be adequately representative. Considering that an adult biopsy sample corresponds to a fraction of just 1/50,000^th^ of the entire liver, a biopsy specimen would seem to be insufficient in diseases such as viral hepatitis, where the liver changes may be unevenly distributed. At present, the most common indication for LB occurs in the setting of chronic viral hepatitis where biopsy is performed to grade and stage histological disease.[44] Therefore, the question that needs to be addressed is whether the sample size affects the histological assessment of chronic hepatitis in terms of grade and stage.

Studies have shown that LB performed with a single pass can miss the diagnosis of cirrhosis in 20%-50% of patients.[4,6,45–48] It has been previously suggested that even a biopsy length of 4 cm may not be the perfect “gold standard,” which is examination of the entire liver or at least a sample longer than 10 cm.[9] Various studies have evaluated the role of LB specimen size that would provide a representative sample for accurate disease estimation.[49–51] A specimen at least 1.5 cm long is needed for an acceptable accuracy in the diagnosis of chronic hepatitis, but larger biopsy samples are mandated when cirrhosis is suspected.[50] The role of biopsy size was further quantified when it was ascertained that diagnostic accuracy depended on the number of complete portal tracts within the biopsy samples. Nevertheless, the number of complete portal tracts required for adequacy of disease differentiation is controversial, with different investigators advocating varying number of portal tracts, ranging from 6 to 11.[44,50,51]

Colloredo *et al*[51] evaluated the effect of core length and diameter on the grading and staging of chronic viral hepatitis. Similar to previous studies,[49,50] the methodology consisted of progressively reducing the length and width of the original samples, which were all at least 2.5-3 cm long. These studies provided robust evidence that both the length and the diameter of the biopsy core affect the grading and staging, and that examining shorter and thinner samples leads to an underestimation of disease severity. Disease activity and fibrosis were underestimated in thin biopsies (i.e., 1 mm wide) regardless of the length of the biopsy, suggesting that the main problem lies in the lower number of complete portal tracts in the smaller samples. The same authors[51] further demonstrated that 11-15 complete portal tracts was the critical number below which disease grade and stage were significantly underestimated, and that a liver biopsy 2 cm long and 1.4 mm wide guaranteed this number of portal tracts in 94% of cases. One recent study using computer-generated modeling estimated that a 2.5-cm biopsy sample yielded an error rate of 25% and that optimal results were obtained with specimens measuring 4 cm.[9] Thus, it is now clear that the four to six portal tracts requirement frequently used by pathologists in clinical practice as well as research protocols, is not sufficient for grading and staging.

In addition, in clinical practice few LB specimens reach the desired length of the biopsy specimen. This also seems to be true in clinical research. A prospective French study revealed that even when performed by an experienced practitioner, about 84% of biopsy samples are smaller than 2 cm.[52] A recent systematic review of 32 studies incorporating 10,027 LB specimens by Cholongitas *et al*[53] reported that the mean±SD length and number of portal tracts were 17.7±5.8 mm and 7.5±3.4 mm, respectively. In this review comprising all documented series of percutaneous LB in the literature, the biopsy specimens had an average length and number of portal tracts well below the published minimum sample size requirements[9,51] in more than half the cases. Since multiple passes would be required to obtain a minimum specimen length of 2 cm, it may potentially increase the complication rate which in turn is based on needle size and number of passes.[54–57] Rocken *et al*[58] demonstrated that irrespective of the method used, LB resulted in an insufficient sample size in a significant proportion of patients. The study showed that only 42% of LB samples with a large 17-gauge needle contained 10 or more portal tracts. Therefore, a minimum requirement for a routine LB specimen to be of 2 cm length could be unrealistic and hazardous for the patient on one hand; on the other hand, the realization that inadequate samples are unreliable would make LB histopathologic examination irrelevant at best and dangerous at worst.

Studies have also shown that differences in grading and staging arise in the setting of different sites of biopsy, suggesting that a random sample may not necessarily reflect damage to the liver as a whole. In a study by Regev *et al*[4] 124 patients with CHC underwent LB of the right and left hepatic lobes during laparoscopy. The comparison between right and left lobes showed a 2-point difference (Scheuer) in grade in 1.6% and a 1-point discordance in 24.2%. As for the stage, discordance in fibrosis scores was observed in 33% of cases. In 2003, Siddique *et al*[59] reported a high variability in the samples amounting to 69% and 62% for activity and fibrosis, respectively. This study analyzed 29 paired biopsies using the Knodell histological activity index, where 69% showed discordance in grade ≥2, and 34.5% revealed a discordance ≥4; the difference in fibrosis score was ≥1 in 38% of cases and ≥2 in 21%. Thus, these findings emphasize that histologic findings may vary according to the site of LB amounting to under-or over-representation of the underlying grade and stage of disease when biopsies are obtained from one lobe only, as is the common practice.

### Variability of histopathologic interpretation

Grading and staging of liver disease are essentially subjective. Several studies have evaluated the interobserver and intraobserver variability in the histologic and pathologic diagnosis of liver fibrosis based on biopsy specimens.[4,60–65] Staging scores for fibrosis such as the METAVIR, Ishak and Scheuer systems were created to standardize the evaluation of liver biopsies to minimize observer variation.[66–68] Although not as great as the errors attributed to sampling variability, interpreter errors may account for 15%–33% of variability[4,9,62] in staging of fibrosis, and 10% of grading of necroinflammation.[4,60] A recent systematic review evaluating observer variation in pathologic scoring systems of LB showed that the widely used Knodell scoring system had a less-than-optimal agreement for grading of liver disease.[53] While the published literature evaluating observer variation in LB interpretation is limited, its scope as a potential confounder to disease stratification is huge. In clinical practice, we frequently encounter the problem of inter-and intraobserver variation and believe that the published literature only represents a small percentage of actual occurrences.

Furthermore, diagnostic errors made by nonspecialist pathologists were reported in more than 25% of patients undergoing LB at academic centers.[69,70] Another study evaluated the rate of concordance between academic hepatopathologists and community pathologists and found that there was 50% interobserver agreement between the pathologists, whereas the community pathologist understaged fibrosis by 73% in patients with chronic hepatitis C virus.[71] This suggests that potentially treatable patients may not receive proper treatment. And, since it is unrealistic to expect the availability of such specialist pathologists in every center performing LB, we can only suspect that the accuracy of the test would be vastly compromised, especially in nonacademic centers.

Lastly, categorization of the extent of inflammation and fibrosis is complicated by the complexity of liver histology scoring systems. These scoring systems, although describing the same histologic parameter, allocate distinctly different numerical scores within different scoring systems. It is also not uncommon that different pathologists within the same institution would not be familiar with the same scoring systems. However, since these scores are not wholly interconvertible or superimposable, a clinician would potentially have to be acquainted with all scoring systems in order to properly interpret histology reports. It must be also noted that the Knodell and Ishak scoring systems[66,72] along with a similar scoring system for steatohepatitis,[73] are not highly reproducible, being only appropriate for statistical analysis of large cohorts of patients in clinical trials.

## NONINVASIVE ASSESSMENT OF LIVER HISTOLOGY

### Ease of performing noninvasive assessment

Noninvasive tests are relatively easy to perform and by extension become easily reproducible. This aspect of noninvasive markers makes them ideally suitable for liver histologic assessment [Table 1]. Moreover, since the clinical course of chronic liver diseases is significantly dependent on the progression rate and the extent of fibrosis, the monitoring of this course with periodic liver histologic assessments is imperative in the overall assessment of the disease.

Simple numeric scores or values as representative of an underlying disease process are intuitively more appealing than the more complex descriptive or semiquantitative scoring methods that are inherent to liver histology assessments. The commonly used noninvasive markers of liver disease utilize a combination of simple biochemical, hematological and demographical parameters. These include laboratory-based tests such as ±2-macroglobulin, total bilirubin, gammaglutamyl transpeptidase (GGT), apolipoprotein A1, haptoglobin, ALT, AST, platelets, age, sex and weight. A composite of various tests calculated according to a patented formula given online, or simple ratios between different parameters, offer easily readable mathematical scores that help distinguish between different levels of histologic disease.[74] Similarly, transient elastography renders simple numerical values in order to distinguish between different stages of fibrosis. For instance, recommended cut-off values for F2, F3 and F4 fibrosis in CHB are 7.2 kilopascals (kPa) (positive predictive value [PPV]=80%, and negative predictive values [NPV]=73%), 8.1–8.4 kPa (PPV=65%–77%, NPV=84%–95%) and 9–11 (PPV=38%–57%, NPV=98%–99%), respectively.[75] Values less than 7 kPa suggest absent or minimal fibrosis.[76]

Training for clinician utilization of transient elastography (FibroScan) is achieved in a simple training schedule extending over a few hours. The ultrasonography-based machine utilizes liver stiffness measurements (10 shots) that are each obtained over duration of few seconds. After rapid training where a minimum experience of 50 shots is recommended, FibroScan provides a reasonable performance for the diagnosis of fibrosis that is not influenced substantially by any other feature.[77] These results emphasize that FibroScan may be used even in nonspecialized units.

### Accuracy of noninvasive markers

Initially, simple noninvasive indexes, such as AST/ALT ratio, platelet count, age-platelet index and APRI were evaluated and found to have moderate diagnostic accuracy for the prediction of significant fibrosis or cirrhosis.[14,78,79] APRI, which is the more accurate of these simple indexes, was reported to provide a moderate to high degree of accuracy (55%-80% agreement with liver biopsy) in identifying the presence of significant fibrosis and cirrhosis in patients with chronic hepatitis C or B.[79,80]

More recently, the next generation of noninvasive markers was developed resulting from multivariate analysis models. These evolved from the basic premise that these markers had to be simple, practical and reasonably accurate in predicting liver fibrosis (85%-95% agreement with liver biopsy).[52,81–83] Table [2] shows a list of the common noninvasive markers of liver histological assessment. Among these, FibroTest is the most widely tested index, and has been validated in several groups of patients with CHB or CHC.[52,81–83] In addition, FibroTest has also been shown to predict the severity of necroinflammation (ActiTest) with the addition of aminotransferase levels.[52,82,83] Transient elastography or FibroScan (Echosens, Paris, France), has shown 85%-90% agreement with liver biopsy for the prediction of significant fibrosis or cirrhosis.[84] In fact, the combined application of FibroScan and FibroTest was suggested to offer the best performance for the assessment of fibrosis in CHC patients with areas under the receiver operator curve (ROC) of 0.88 for ≥F2, 0.95 for ≥F3 and 0.95 for F4.[84]

**Table 2:** Comparison of liver biopsy and noninvasive markers of liver histology

**Parameter**	**Noninvasive markers**	**Liver biopsy**
Cost	$150-450	$1000-2200
Ease of testing	Phlebotomy, assay materials	Operator, pathology laboratory, pathologist
Time for results	Dependent on clinical laboratory: <2 hours	Dependent on pathology laboratory: 24-72 hours minimum
False negatives	Varies per test: up to 25%	Up to 30% (sampling variability)
Complications	Negligible	Significant bleeding, pain, viscus perforation, death
Accuracy	Varies: ~80%	80%
Contraindications	Known conditions with high rates of false	Bleeding diathesis/coagulopathy, ascites, uncooperative patient,
	positivity	extrahepatic biliary obstruction, morbid obesity
Specimen adequacy	Adequacy guaranteed	At least 1.5 cm in length with 6–8 portal tracts; ~50%
Observer variability	Machine-generated results	Expertise-dependent; 10% for inflammation, up to 30% for fibrosis
Monitoring	Can be repeatedly performed over time to monitor disease	Vastly limited due to invasiveness and risks; patient acceptance low

In a recent systematic review of eight CHC studies incorporating 1503 subjects assessing FibroTest, the sensitivity, specificity and area under the summary ROC curve were reported as 47%, 80% and 0.81, respectively, for significant fibrosis (F2-4).[85] The same review evaluated four studies (504 subjects) reporting the utility of FibroScan, and reported the sensitivity, specificity and area under the summary ROC curve as 64%, 87% and 0.83, respectively, for fibrosis (F2–4). Similarly, a recent study in CHB patients calculated the area under the ROC curve for three different fibrosis stage thresholds (in relation to F0-1).[86] The reported area under the ROC curves for ≥F2, ≥F3 and F4 fibrosis were 0.81, 0.93 and 0.93, respectively. Halfon *et al*[87] showed that FibroTest–ActiTest can distinguish between little or no fibrosis (F0–1) and bridging fibrosis (≥F2) with a specificity of 72%. More significantly, in a prospective study, Poynard *et al*[88] estimated that 18% of discordances between FibroTest–ActiTest and histology were attributable to biopsy failure and just 2% to test failure. Thus, these studies demonstrate that FibroTest–ActiTest and FibroScan have excellent utility for the identification of CHC-and CHB-related minimal and advanced fibrosis.

In addition to demonstrating accuracy in viral hepatitis, noninvasive markers have also been validated in patients with alcoholic and nonalcoholic fatty liver disease (NAFLD). Poynard *et al*[89] have demonstrated the utility of SteatoTest/ NashTest, a biomarker combining FibroTest–ActiTest with body mass index, cholesterol, triglycerides and glucose, in subjects with NAFLD and showed excellent diagnostic accuracy. Likewise, FibroScan has been validated for biliary fibrosis in patients with cholestatic liver diseases.[90,91] Thus, a wide variety of liver diseases have been assessed by noninvasive markers and their adequate validations performed.

### Cost-effectiveness of the procedure

In a French survey, general anesthesia is reported to be used in 11% of LB cases, benzodiazepine in 16% and atropine with benzodiazepine in 15%.[57] In the United States, 54% of gastroenterologists/hepatologists and 96% of radiologists use conscious sedation.[92] LB requires admission to the hospital, and the administration of conscious sedation requires a high level of hemodynamic monitoring and skilled nursing staff for safe post-biopsy care. Another survey of 260 randomly selected members of the American Association for the Study of Liver Diseases (AASLD) showed that 62% utilized an ultrasonographer to mark the biopsy site, while 18% had the biopsy performed by the radiologist with real-time ultrasound guidance.[93] Because of the monitoring, processing and interpretation required, the cost of percutaneous LB is significant. An LB at most hospitals in the United States costs approximately $2200,[94] while in Britain the average cost for an inpatient biopsy is $1000[95] and in Australia is $1032.[96] This cost does not include the additional expenses of hospitalization and treatment for patients who develop complications of the procedure. The cost of noninvasive markers, FibroTest–ActiTest and FibroScan, although variable, is vastly lower than LB, amounting to an estimated cost of $150-450 per test.[97]

## CONCLUSION

The ideal test for liver histologic assessment should have high sensitivity and specificity, be relatively inexpensive, incur minimal risk for the patient and be convenient to perform with reproducible and easily interpreted results. LB entails significant complications toward liver histologic assessment. It also suffers serious shortcomings in diagnostic accuracy. A large liver sample size is required to achieve an ideal diagnostic accuracy, which is clinically infeasible and even dangerous to pursue. On the other hand, a number of noninvasive biomarkers have evolved, each with an impressive range of diagnostic certainty approaching that achieved with LB. These pose no danger to the patient, are reproducible, and yet easily interpretable. Invasive assessment of the liver can no longer be cited as a “gold standard,” and at best can only be considered as an imperfect standard. Neither LB nor any single alternative option represents an absolute assessment of liver disease.
